# Endoscopic resection of a giant long-pedunculated Brunnerʼs gland hyperplasia in the duodenum

**DOI:** 10.1055/a-2814-5981

**Published:** 2026-03-11

**Authors:** Tingting Cao, Rui Xie, Xiaozhong Yang, Honggang Wang, Weijie Dai

**Affiliations:** 1 91596 Department of Gastroenterology, The Affiliated Huaiʼan No. 1 Peopleʼs Hospital of Nanjing Medical University, Huaiʼan, China

A 69-year-old woman was admitted to the hospital due to recurrent melena for 5 days. Her medical history included diabetes and cerebral infarction, and she was regularly on oral aspirin and glipizide. Laboratory tests upon admission revealed a hemoglobin level of 51 g/L. Gastroscopy indicated a long-pedunculated elevated lesion originating from the first portion of the duodenum, extending into the second portion with a length of approximately 6 cm. An ulcer was observed on the top of the lesion, accompanied by oozing ( Fig. 1 **a, b** ). Given the high bleeding risk from the thick peduncle, prophylactic measures were necessary. After evaluating alternatives: epinephrine injection (transient effect), pre-clipping (the risk of incomplete closure or slippage), and endoloop ligation, we chose the latter for definitive circumferential mechanical occlusion. After resection, titanium clips were applied to close the wound and secure the endoloop to prevent slippage ( Video 1 ). All procedures were performed using an Olympus Evis X1 endoscopy system, with electrosurgical resection conducted via an ERBE VIO 300D generator using Endocut Q (effect 3, duration 2, and interval 4) for cutting and forced coagulation (effect 2 and 40 W) for hemostasis. No immediate or delayed complications, such as bleeding and perforation, occurred. Histopathological examination confirmed the diagnosis of duodenal Brunner’s gland hyperplasia ( Fig. 1 **c** ). The patient fasted for 24 hours, received proton pump inhibitor therapy, and was discharged on postoperative day 4. At the 1-month follow-up, she remained asymptomatic.

**Fig. 1:**
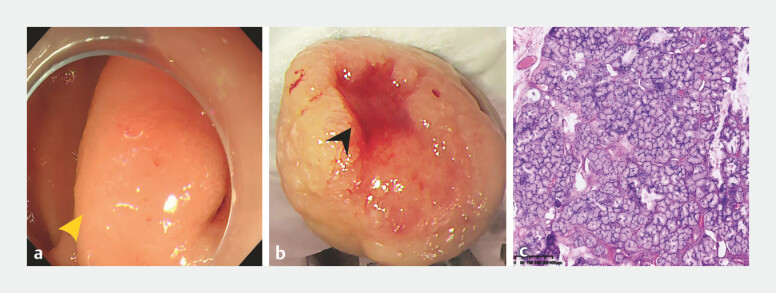
**a, b** Gastroscopy revealed a long-pedunculated elevated lesion (yellow arrow). An ulcer with active oozing was observed on the surface of the lesion (black arrow). **c** Histopathological examination revealed numerous hyperplastic Brunner’s glands within the submucosa (scale bar, 400 μm).

Endoscopic resection of a giant pedunculated duodenal Brunner’s gland hyperplasia.Video 1

Brunner’s gland hyperplasia refers to the benign hyperplasia of glands in the duodenal mucosa or submucosa, and its incidence is extremely low 1 . It is usually asymptomatic and often discovered incidentally. In this case, the long-term aspirin use may have precipitated ulceration and bleeding on the surface of the Brunner’s gland hyperplasia. For such lesions, endoscopic resection represents a safe and effective therapeutic option.

Endoscopy_UCTN_Code_TTT_1AO_2AG_3AB

## References

[1] Bhatti S Alghamdi M Omer E Brunner’s Gland Hyperplasia: A Massive Duodenal Lesion Cureus 2020 12 e7542 10.7759/cureus.7542 32377490 PMC7198079

